# Posterior cingulate cortex targeted real‐time fMRI neurofeedback recalibrates functional connectivity with the amygdala, posterior insula, and default‐mode network in PTSD

**DOI:** 10.1002/brb3.2883

**Published:** 2023-02-15

**Authors:** Jonathan M. Lieberman, Daniela Rabellino, Maria Densmore, Paul A. Frewen, David Steyrl, Frank Scharnowski, Jean Théberge, Richard W. J. Neufeld, Christian Schmahl, Rakesh Jetly, Sandhya Narikuzhy, Ruth A. Lanius, Andrew A. Nicholson

**Affiliations:** ^1^ Department of Psychiatry and Behavioural Neurosciences McMaster University Hamilton Ontario Canada; ^2^ Imaging, Lawson Health Research Institute London Ontario Canada; ^3^ Department of Neuroscience Western University London Ontario Canada; ^4^ Department of Psychiatry Western University London Ontario Canada; ^5^ Department of Psychology Western University London Ontario Canada; ^6^ Department of Cognition, Emotion, and Methods in Psychology University of Vienna Vienna Austria; ^7^ Department of Medical Biophysics Western University London Ontario Canada; ^8^ Department of Diagnostic Imaging St. Joseph's Healthcare London Ontario Canada; ^9^ Department of Psychology University of British Columbia, Okanagan Kelowna British Columbia Canada; ^10^ Department of Psychosomatic Medicine and Psychotherapy Central Institute of Mental Health Mannheim Heidelberg University Heidelberg Germany; ^11^ The Institute of Mental Health Research University of Ottawa, Royal Ottawa Hospital Ontario Canada; ^12^ Homewood Research Institute Guelph Ontario Canada; ^13^ Atlas Institute for Veterans and Families Ottawa Ontario Canada; ^14^ School of Psychology University of Ottawa Ottawa Canada

**Keywords:** default mode network, fMRI neurofeedback, posterior cingulate cortex, posttraumatic stress disorder

## Abstract

**Background:**

Alterations within large‐scale brain networks—namely, the default mode (DMN) and salience networks (SN)—are present among individuals with posttraumatic stress disorder (PTSD). Previous real‐time functional magnetic resonance imaging (fMRI) and electroencephalography neurofeedback studies suggest that regulating posterior cingulate cortex (PCC; the primary hub of the posterior DMN) activity may reduce PTSD symptoms and recalibrate altered network dynamics. However, PCC connectivity to the DMN and SN during PCC‐targeted fMRI neurofeedback remains unexamined and may help to elucidate neurophysiological mechanisms through which these symptom improvements may occur.

**Methods:**

Using a trauma/emotion provocation paradigm, we investigated psychophysiological interactions over a single session of neurofeedback among PTSD (*n* = 14) and healthy control (*n* = 15) participants. We compared PCC functional connectivity between regulate (in which participants downregulated PCC activity) and view (in which participants did not exert regulatory control) conditions across the whole‐brain as well as in a priori specified regions‐of‐interest.

**Results:**

During regulate as compared to view conditions, only the PTSD group showed significant PCC connectivity with anterior DMN (dmPFC, vmPFC) and SN (posterior insula) regions, whereas both groups displayed PCC connectivity with other posterior DMN areas (precuneus/cuneus). Additionally, as compared with controls, the PTSD group showed significantly greater PCC connectivity with the SN (amygdala) during regulate as compared to view conditions. Moreover, linear regression analyses revealed that during regulate as compared to view conditions, PCC connectivity to DMN and SN regions was positively correlated to psychiatric symptoms across all participants.

**Conclusion:**

In summary, observations of PCC connectivity to the DMN and SN provide emerging evidence of neural mechanisms underlying PCC‐targeted fMRI neurofeedback among individuals with PTSD. This supports the use of PCC‐targeted neurofeedback as a means by which to recalibrate PTSD‐associated alterations in neural connectivity within the DMN and SN, which together, may help to facilitate improved emotion regulation abilities in PTSD.

## 1 INTRODUCTION

Posttraumatic stress disorder (PTSD) is a severely debilitating psychiatric condition that may develop among individuals in the aftermath of trauma and includes symptoms of trauma‐related intrusive recollections and flashbacks, hyperarousal/hyperreactivity, avoidance behaviors, and negative alterations in cognitions and mood (American Psychiatric Association, 2022). Concerningly, a significant proportion of individuals with PTSD have difficulty tolerating and/or do not experience clinically significant symptom reductions from gold‐standard psychotherapeutic and pharmacotherapeutic treatments (Bradley et al., 2005; Haagen et al., 2015; Kline et al., 2018; Krystal et al., 2017; Ravindran & Stein, 2009; Stein et al., 2006). As a result, many individuals remain burdened with persistent symptoms. In PTSD, it is well documented that there are strong associations between symptom presentations and multiple functional disruptions in the brain (Fenster et al., 2018; Lanius et al., 2015; Tursich et al., 2015). As such, emerging research supports the notion that recalibrating PTSD‐associated functional disruptions within neural circuits constitutes a promising treatment approach that may complement existing psycho‐ and pharmacotherapies (Chiba et al., 2019; Koek et al., 2019; Nicholson et al., 2021; Nicholson et al., 2020; Panisch & Hai, 2020; Rogel et al., 2020).

### 1.1 Disrupted functional networks in PTSD

Recent functional neuroimaging research has found that many neuropsychiatric disorders, including PTSD, are associated with alterations within large‐scale functional brain networks, that is, intrinsic connectivity networks (ICNs) (Akiki et al., 2018; Breukelaar et al., 2021; Daniels et al., 2010; Fenster et al., 2018; Lanius et al., 2010; Nicholson et al., 2020; Sripada et al., 2012; Tursich et al., 2015; Weng et al., 2019). Three core ICNs—the default mode network (DMN), the central executive network (CEN), and the salience network (SN)—appear to be particularly important for cognitive function and dysfunction, wherein altered connectivity has been implicated in a wide range of psychopathologies (Albert et al., 2019; Gong et al., 2019; Krause et al., 2017; Li et al., 2018; McFadden et al., 2014; Menon, 2011; Menon, 2020; Otti et al., 2013; Shao et al., 2018). In PTSD, there is an extensive knowledge base linking disruptions within the DMN and SN to specific PTSD symptoms (Fenster et al., 2018; Lanius et al., 2015; Nicholson et al., 2021; Rabellino et al., 2015; Ross & Cisler, 2020), where critically, emerging evidence suggests that neurofeedback may be an effective treatment option for recalibrating such neural network disruptions (Bell et al., 2019; Gerin et al., 2016; Kluetsch et al., 2014; Misaki et al., 2019; Misaki et al., 2018; Nicholson et al., 2021; Nicholson et al., 2020; Nicholson et al., 2018; Nicholson et al., 2016; Nicholson et al., 2016; Zotev et al., 2018).

More specifically, the DMN is comprised of several midline brain regions, including the posterior cingulate cortex (PCC), the precuneus, and the medial prefrontal cortex (mPFC), which together subserve functions of self‐referential processing, social cognition, and autobiographical memory (Frewen et al., 2020; Qin & Northoff, 2011). Individuals with PTSD have repeatedly been found to display decreased coupling between major DMN nodes at rest, where the magnitude of decrease has been associated with greater PTSD symptom severity (Bluhm et al., 2009; di et al., 2012; Koch et al., 2016; Sripada et al., 2012). Among individuals with PTSD, functional disruptions in the DMN have been associated with traumatic/negative autobiographical memories, as well as distorted and fragmented self‐referential processing (Akiki et al., 2017; Bluhm et al., 2009; Daniels et al., 2010; Fenster et al., 2018; Lanius et al., 2015; Tursich et al., 2015). Graph theoretical analyses have found spared connectivity within posterior DMN nodes (i.e., PCC, precuneus), relative to decreased connectivity within anterior DMN nodes, including the mPFC, among patients with PTSD during rest (Akiki et al., 2018; Holmes et al., 2018; Kennis et al., 2016; Shang et al., 2014). On the other hand, during working memory tasks, individuals with PTSD have been found to display maladaptive increased connectivity of the PCC with other DMN areas, as compared to increased connectivity with CEN and SN regions observed in healthy individuals (Daniels et al., 2010). This may reflect the difficulty faced by patients with PTSD in disengaging attention from self‐referential processing to focus on more task‐relevant stimuli (Aupperle et al., 2012). Furthermore, there is a substantial body of literature establishing enhanced activation within the PCC and other DMN regions among individuals with PTSD during the reliving and reexperiencing of trauma‐related autobiographical memories (Awasthi et al., 2020; Fenster et al., 2018; Frewen et al., 2011; Hopper et al., 2007; Liberzon & Abelson, 2016; Mickleborough et al., 2011; Ramage et al., 2013; Thome et al., 2020). Indeed, findings of enhanced DMN recruitment in response to trauma reminders is not surprising given that individuals living with PTSD can often report feeling as though their sense‐of‐self is inextricably fused with their experiences of trauma (Fenster et al., 2018; Frewen et al., 2011; Lanius et al., 2020; Liberzon & Abelson, 2016; Terpou et al., 2019; Terpou et al., 2020; Thome et al., 2020).

The SN, with core nodes in the amygdala, insula, and dorsal anterior cingulate cortex (dACC), is involved in environmental monitoring, interoceptive processing, autonomic regulation, and approach/avoidance behaviors (Dosenbach et al., 2007; Gogolla, 2017; Modinos et al., 2009; Namkung et al., 2017; Seeley et al., 2007; Sridharan et al., 2008). Regions of the SN are also critically involved in the innate alarm system, a network of brain regions that together enable the rapid detection and response to threatening environmental stimuli via the integration of emotion and sensory information, which has critical implications for chronic stress exposure and PTSD (Lanius et al., 2017; Szeszko & Yehuda, 2019). Among individuals with PTSD, alterations in resting‐state SN activity have been associated with symptoms of hyperarousal, hypervigilance, avoidance, and altered interoception (Akiki et al., 2017; Harricharan et al., 2020; Koch et al., 2016; McCurry et al., 2020; Nicholson et al., 2020; Rabinak et al., 2011; Sripada et al., 2012; Tursich et al., 2015; Yehuda et al., 2015). Specifically, studies have found that individuals with PTSD exhibit increased coupling of the anterior insula within the SN, as well as reduced connectivity between the SN and the dorsolateral prefrontal cortex (dlPFC) which is involved in emotion regulation (Harricharan et al., 2020; Jeong et al., 2019; Koch et al., 2016; Lanius et al., 2015; Nicholson et al., 2020; Sripada et al., 2012). With regard to the amygdala, a limbic region centrally involved in emotion generation/processing and the innate alarm system, hyperactivity has been repeatedly demonstrated in association with PTSD psychopathology (Aghajani et al., 2016; Birn et al., 2014; Etkin et al., 2011; Fenster et al., 2018; Fitzgerald et al., 2018; Koch et al., 2016; Lanius et al., 2015; Lanius et al., 2010; Mickleborough et al., 2011; Patel et al., 2012; Schulze et al., 2019; Yehuda et al., 2015). Indeed, the role of altered SN activity in PTSD is unsurprising given that many PTSD symptoms (e.g., hyperarousal, hypervigilance, irritability, aggression) often involve maladaptive coding of salience and thus misassignment of the appropriate degree of imminence and threat to external stimuli (Szeszko & Yehuda, 2019). Interestingly, in a narrative review of MRI studies that attempted to predict PTSD treatment response to psychotherapies (i.e., cognitive behavioral therapy and exposure‐based therapies), it was found that better responses following psychotherapeutic treatment were associated with reduced amygdala and anterior insula activity along with increased ACC activity (Szeszko & Yehuda, 2019), thus indicating that recalibrating altered SN connectivity may be a critical path towards improvement of PTSD symptoms. Taken together, it is apparent that altered connectivity patterns within both the DMN and SN are highly associated with PTSD symptoms. For this reason, interventions that restore DMN and SN functioning may represent a promising treatment avenue for reducing PTSD symptomatology (Koek et al., 2019; Nicholson et al., 2020; Szeszko & Yehuda, 2019). Of importance, emerging evidence from neurofeedback studies in PTSD suggest that it may be effective in recalibrating aberrant DMN and SN dynamics and reducing associated symptoms (Bell et al., 2019; Gerin et al., 2016; Kluetsch et al., 2014; Misaki et al., 2019; Misaki et al., 2018; Nicholson et al., 2021; Nicholson et al., 2020; Nicholson et al., 2018; Nicholson et al., 2016; Nicholson et al., 2016; Zotev et al., 2018).

### 1.2 Restoring DMN and SN connectivity in PTSD with neurofeedback

Neurofeedback is an emerging adjunctive treatment approach that has been investigated across a wide range of psychiatric disorders (Li et al., 2013; Linden et al., 2012; Mehler et al., 2018; Schoenberg & David, 2014; Young et al., 2017), including PTSD (Bois et al., 2021; Chiba et al., 2019; Gerin et al., 2016; Kluetsch et al., 2014; Misaki et al., 2019; Misaki et al., 2018; Nicholson et al., 2021; Nicholson et al., 2020; Nicholson et al., 2018; Nicholson et al., 2016; van der Kolk et al., 2016; Weaver et al., 2020; Zotev et al., 2018; Zweerings et al., 2018). During neurofeedback, neural activity is measured and presented to participants in real‐time, thus allowing them to noninvasively self‐regulate brain activity (Sitaram et al., 2017). As such, individuals with PTSD can learn to regulate disrupted brain activity that is associated with the manifestation and maintenance of their symptoms. Importantly, recalibrating activity within main ICN hubs may help restore optimal interactions within‐ and between‐ICNs. As altered connectivity within the DMN and SN are highly associated with PTSD symptoms, utilizing neurofeedback to restore DMN and SN connectivity may be a promising approach for reducing PTSD symptoms. Importantly, preliminary evidence suggests that neurofeedback may be effective in recalibrating aberrant DMN and SN dynamics and reducing PTSD symptoms (Kluetsch et al., 2014; Nicholson et al., 2021; Nicholson et al., 2020; Nicholson et al., 2018; Nicholson et al., 2016; Nicholson et al., 2016) via homeostatic neuroplasticity (Bois et al., 2021; Ros et al., 2014; Sitaram et al., 2017; Nicholson et al., 2023) and, as such, merits further investigation.

Previous electroencephalography (EEG) neurofeedback studies have been conducted in PTSD, including single‐session mechanistic studies (Kluetsch et al., 2014; Nicholson et al., 2016) and a 20‐week randomized controlled trial (Nicholson et al., 2020; Nicholson et al., 2023; Shaw and Nicholson et al., 2023), targeting alpha oscillations over the PCC. Alpha oscillations are correlated with activation of the DMN, including the PCC (Clancy et al., 2020; Jann et al., 2009; Mantini et al., 2007), where reduced resting‐state alpha‐rhythms in PTSD are thought to reflect chronic hyperarousal associated with SN hyperactivity and dysregulated DMN dynamics (Abdallah et al., 2017; Clancy et al., 2020; Clancy et al., 2017; Liberzon & Abelson, 2016; Nicholson et al., 2020; Ros et al., 2014; Sitaram et al., 2017). We found previously that a single session of alpha‐based EEG neurofeedback recalibrated DMN and SN resting‐state functional connectivity, which was associated with reduced hyperarousal symptoms (Kluetsch et al., 2014; Nicholson et al., 2016). Similarly, in our randomized controlled trial of alpha‐based EEG neurofeedback, significantly decreased PTSD severity scores in the experimental group were associated with restored patterns of resting‐state connectivity within the DMN (i.e., increased dmPFC connectivity and decreased PCC/precuneus connectivity with the DMN) and SN (i.e., decreased anterior insula connectivity with the SN) (Nicholson et al., 2020). Importantly, at a 3‐month follow‐up, over 60% of experimental group participants no longer met diagnostic criteria for PTSD (Nicholson et al., 2020), a remission rate comparable to currently available psychotherapies and pharmacotherapies for PTSD (Berger et al., 2009; Bradley et al., 2005; Haagen et al., 2015; Krystal et al., 2017; Ravindran & Stein, 2009; Stein et al., 2006). Taken together, these results indicate that regulating PCC/posterior DMN associated brain signals (i.e., alpha rhythms) may be effective in reducing PTSD symptoms and recalibrating altered DMN and SN connectivity among individuals with PTSD (Kluetsch et al., 2014; Nicholson et al., 2020; Nicholson et al., 2016; Nicholson et al., 2023; Shaw and Nicholson et al., 2023). In comparison with EEG neurofeedback, real‐time functional magnetic resonance imaging (fMRI) neurofeedback (rt‐fMRI‐NFB) affords superior spatial resolution and is therefore a critical modality for generating evidence to further elucidate neurophysiological mechanisms underlying treatment success.

Real‐time fMRI‐NFB has been successfully implemented in PTSD populations, where a number of previous studies have targeted regulation of the amygdala (Chiba et al., 2019; Gerin et al., 2016; Misaki et al., 2019; Misaki et al., 2018; Nicholson et al., 2018; Nicholson et al., 2016; Zotev et al., 2018). Indeed, in a previous study by our group, it was shown that among PTSD participants exposed to personalized trauma words, downregulating the amygdala was associated with increased activity and connectivity of the dlPFC and vlPFC, brain regions associated with emotion regulation (Nicholson et al., 2016), a finding that was replicated in other amygdala‐targeted rt‐fMRI‐NFB studies in PTSD (Misaki et al., 2018; Zotev et al., 2018). Moreover, in a network‐based analysis, amygdala downregulation was also found to induce neuroplastic changes within ICNs, including the DMN and SN, over neurofeedback training (Nicholson et al., 2018), an effect similar to those found in EEG‐based neurofeedback investigations (Kluetsch et al., 2014; Nicholson et al., 2020; Nicholson et al., 2016).

Most recently, our group utilized rt‐fMRI‐NFB to train individuals with PTSD and healthy controls to downregulate PCC activity during the same trauma/emotion provocation paradigm in which participants viewed personalized trauma/distressing words (Nicholson et al., 2021). The PCC was targeted for neurofeedback as it shows hyperactivity during the reliving and reexperiencing of trauma memories among individuals with PTSD (Awasthi et al., 2020; Fenster et al., 2018; Frewen et al., 2011; Hopper et al., 2007; Liberzon & Abelson, 2016; Mickleborough et al., 2011; Ramage et al., 2013; Thome et al., 2020). In a previously published analysis, we reported that individuals in the PTSD group showed reduced symptoms of reliving and distress in response to trauma‐related stimuli over neurofeedback training (Nicholson et al., 2021), an effect not observed during the single‐session rt‐fMRI‐NFB study targeting the amygdala (Nicholson et al., 2018; Nicholson et al., 2016). While both groups were able to downregulate their PCC activity with similar success over neurofeedback training, downregulation was associated with unique within‐PTSD group decreases in neural activity in several brain regions including those of the DMN (i.e., bilateral dmPFC, hippocampus) and SN (i.e., amygdala, mid‐cingulate cortex) (Nicholson et al., 2021). Interestingly, these findings add to a growing body of research that highlight the critical involvement of the PCC in emotion regulation, including associations between normalized PCC activity and positive clinical outcomes following psychotherapy (Fresco et al., 2017; Garrett et al., 2019; Scult et al., 2019), as well as positive correlations between PCC‐amygdala connectivity and PTSD symptom severity following trauma exposure (Lanius et al., 2010; Zhou et al., 2012). The PCC is also critical in neural circuits supporting cognitive reappraisal, attentional shifting, and acceptance (Ferri et al., 2016; Kanske et al., 2011; King et al., 2016; Ma et al., 2017; Messina et al., 2021), all of which are key volitional emotion regulation strategies. Notably, however, functional connectivity of the PCC during rt‐fMRI‐NFB has not previously been investigated among individuals with PTSD. In the present analysis, our goal was to further examine how PCC‐targeted fMRI neurofeedback may recalibrate connectivity within large‐scale brain networks. In doing so, we were able to characterize neural mechanisms that might underlie improvements in emotion regulation among PTSD participants that were previously reported by our group (Nicholson et al., 2021) and also evaluate whether findings are convergent with that of previous EEG‐based neurofeedback studies (Kluetsch et al., 2014; Nicholson et al., 2020; Nicholson et al., 2018). Given the accumulation of functional neuroimaging findings of altered DMN and SN activity in PTSD, as well as studies which have demonstrated a recalibration of DMN and SN connectivity as a function of successful neurofeedback treatment for PTSD, examination of functional connectivity between the PCC and key hubs of both networks was of particular interest in this study.

### 1.3 Current study

In the current rt‐fMRI‐NFB study, individuals with PTSD and healthy controls trained to downregulate their PCC during a trauma/emotion provocation paradigm, wherein participants viewed trauma‐related/distressing words. In the present analysis, we utilized the psychophysiological interaction (PPI) method to elucidate within‐ and between‐ group differences in PCC functional connectivity during neurofeedback training. During regulation, we hypothesized that the PCC would display unique functional connectivity to key DMN and SN hubs within the PTSD group. Furthermore, we also expected to observe positive associations between psychiatric symptoms and functional connectivity between the PCC and both DMN and SN hubs.

## 2 METHODS

### 2.1 Participants

Our total sample (*n =* 30) consisted of *n =* 15 participants who met the criteria for a current primary diagnosis of PTSD, as determined by the Clinician‐Administered PTSD Scale (CAPS‐5) (Weathers et al., 2018) and the Structured Clinical Interview for DSM‐5 (SCID) (First, 2015), and *n =* 15 healthy, nontrauma‐exposed controls (see Table 1). One participant in the PTSD group was not included in the analysis as they self‐reported falling asleep in the scanner during the transfer run. Hence, the final sample size was *n* = 14 in the PTSD group and *n* = 15 in the healthy control group. The PTSD and healthy control groups had nonsignificant differences with respect to biological sex. The mean age of PTSD participants was significantly higher than that of healthy control participants. As such, we included age as a covariate in our analyses and, importantly, did not find any significant differences in PCC functional connectivity results. Similarly, given its potential impact on brain connectivity, participant handedness was also included as a covariate in our analyses and was found not to yield any significant differences in PCC functional connectivity. Notably, no study participants reported a significant history of head injuries without loss of consciousness. While all analyses presented in this paper are novel, the imaging data attained from this participant group was previously analyzed in a recently published work (Nicholson et al., 2021).

**TABLE 1:** Demographic and clinical information

	PTSD group (*N* = 14)	Healthy control group (*N* = 15)
Biological sex	6F, 8 M	10F, 5 M
Years of age	49.50 (±5.11)	37.73 (±12.86)
CAPS‐5	43.21 (±8.26)	0 (±0)
BDI	32.14 (±12.55)	1.20 (±2.46)
CTQ	61.50 (±25.84)	31.13 (±8.44)
MDI	87.36 (±28.23)	43.20 (±4.36)
DERS	107.64 (±24.84)	52.80 (±9.03)
Psychotropic medication	10	0
MDD—Current	9	0
MDD—Past	2	0
Other psychiatric conditions—Current	5	0

*Note*: Values in parentheses indicate standard deviation. Other psychiatric conditions include agoraphobia (*N* = 1), panic disorder (*N* = 1), and somatic symptom disorder (*N* = 3). PTSD = Posttraumatic Stress Disorder, CAPS = Clinician‐Administered PTSD Scale, BDI = Beck's Depression Inventory, CTQ = Childhood Trauma Questionnaire (*none or minimal childhood trauma = 25–36, moderate = 56–68, extreme trauma > 72*), MDI = Multiscale Dissociation Inventory, DERS = Difficulties in Emotion Regulation Scale, MDD = Major Depressive Disorder.

Participants were recruited between 2017 and 2019 via clinician referrals, community programs for traumatic stress and posters within the London, Ontario community. All scanning took place at the Lawson Health Research Institute in London, Ontario, Canada. The study was approved by the Research Ethics Board at Western University, Ontario, Canada. All study participants provided written and informed consent and received financial compensation for their participation in this study.

Participants were included in the PTSD group if they had a current primary diagnosis of PTSD as measured by the CAPS‐5 and SCID. PTSD participants currently receiving psychotropic medication were all on a stable dose for at least 1 month prior to their participation in the current study. PTSD participants were excluded from the study if they had ongoing and/or recent (within previous 3 months) alcohol or substance use disorders, suicidal ideations, or self‐injurious behaviors requiring medical attention. Lifetime diagnoses of bipolar or psychotic disorders were additional exclusion criteria for PTSD participants. Control participants were excluded from the study if they had a lifetime diagnosis of psychiatric illness or currently used psychotropic medications. Exclusion criteria for all participants included previous biofeedback treatment, noncompliance with 3T fMRI safety guidelines, untreated medical conditions, pregnancy, previous head injury with loss of consciousness, and neurological or pervasive developmental disorders. Please see the supplementary materials section (Table S1) for information pertaining to the history of trauma exposure in each group.

In this study, all participants completed several clinical assessments prior to scanning. These assessments included Beck's Depression Inventory (BDI) (Beck et al., 1997), the Childhood Trauma Questionnaire (CTQ) (Bernstein et al., 2003), Difficulties in Emotion Regulation Scale (DERS) (Gratz & Roemer, 2003), and the Multiscale Dissociation Inventory (MDI) (Briere et al., 2005). Additionally, after each of the four fMRI neurofeedback runs, participants completed the Response to Script Driven Imagery (RSDI) Scale (Hopper et al., 2007), consisting of the following symptom subscales: reliving, distress, physical reactions, dissociation, and numbing.

### 2.2 Neurofeedback paradigm

**FIGURE 1:**
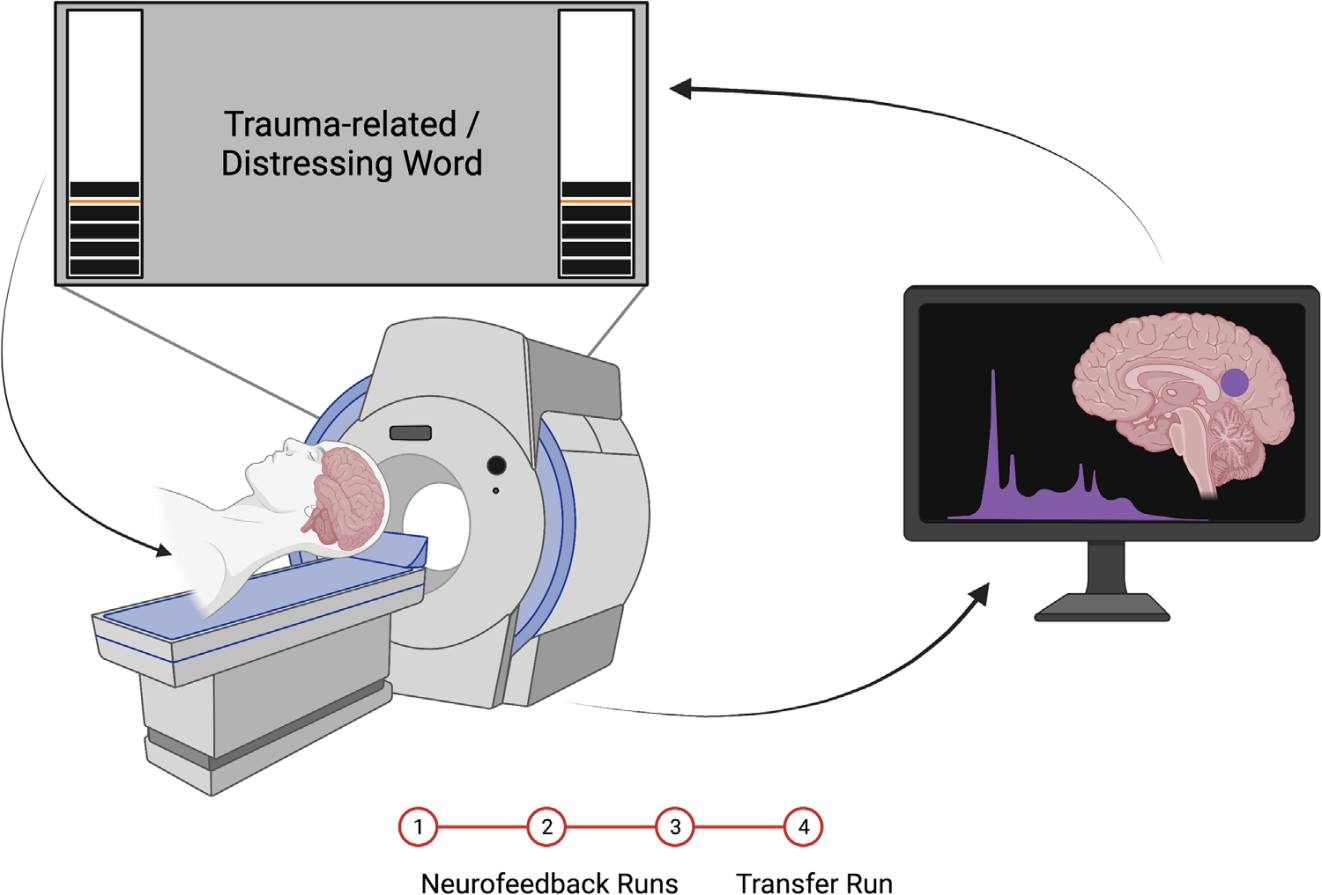
Depiction of the rt‐fMRI‐NFB set‐up. While participants were inside the scanner, they were presented with a neurofeedback signal in the form of a virtual thermometer that increased/decreased in response to fluctuating activity within the neurofeedback target region (PCC). Participants completed three neurofeedback training runs, followed by a transfer run, in which they were not presented with the neurofeedback signal. Figure reproduced with permission from Nicholson et al. (2021).

This study used an experimental protocol and neurofeedback paradigm as described in our previously published work (Nicholson et al., 2021; Nicholson et al., 2018; Nicholson et al., 2016) (see Figure 1). During neurofeedback training runs, participants were shown a neurofeedback signal corresponding to activation in their PCC. The neurofeedback signal was made to appear as two identical thermometers on either side of a screen projected to participants while they were inside the scanner. The bars on the thermometer increased/decreased in correspondence to changing BOLD signal from the PCC target region. Each segment within the thermometer corresponded to a 0.2% activation change in the PCC, with a maximal increase range of 2.8% and a maximal decrease range of 1.2% from baseline activation (Nicholson et al., 2021; Nicholson et al., 2016; Paret et al., 2014; Paret et al., 2016). At the onset of each trial, the mean of the preceding four data points was taken as the baseline and displayed to participants as an orange line on the thermometer (Nicholson et al., 2021; Nicholson et al., 2016; Paret et al., 2014; Paret et al., 2016). Participants were not provided with specific instructions on regulation strategies. Rather, participants were told that they would be “regulating an area of the brain related to emotional experience.” Participants were instructed to focus their vision on the word for the entire duration of each condition, while using their peripheral vision to monitor the thermometers. Before the first run, participants were informed about the temporal delay in neurofeedback signal due to the BOLD signal delay.

Our neurofeedback protocol consisted of three conditions: regulate, view, and neutral. In the regulate condition, participants were instructed to decrease the neurofeedback signal, corresponding to activity in the PCC seed region, while viewing a personalized trauma‐related word (PTSD group) or a matched distressing word (healthy control group). In the view condition, participants viewed the chosen words but were instructed to respond naturally and not attempt to change the neurofeedback signal. In the neutral condition, participants in both groups viewed personalized neutral words and were instructed to respond naturally. Participants selected personalized trauma/distressing words (*n* = 10) and neutral words (*n* = 10) under the guidance of a trauma‐informed clinician. Words chosen by PTSD participants were related to their individual experiences of trauma, whereas control participants selected words associated with their most stressful life experience. All chosen words were matched on subjective units of distress to control for between‐subject/group variability. Stimuli were presented using the Presentation software, Neurobehavioural Systems (Berkeley, California). The experimental design included three consecutive neurofeedback training runs, followed by a single transfer run. The transfer run was identical to the training runs except participants did not receive any neurofeedback signal. Each run lasted 9 min and included 15 trials (five per condition). The timing for all trials was as follows: 2 s for instructions, followed by 24 s for individual conditions, and then a 10 s implicit resting state in which participants viewed an intertrial fixation cross. All trials were counterbalanced.

### 2.3 Real‐time signal processing for neurofeedback

To present real‐time PCC neural activation to participants via thermometer display, anatomical scans were imported into BrainVoyager (version QX2.4; Brain Innovations, Maastrict, Netherlands), then skull‐stripped and transformed into Talairach space. Subsequently, normalization parameters were input into TurboBrainVoyager (TBV) (version 3.0, Brain Innovations, Maastricht, Netherlands). A 4‐mm full‐width‐half‐maximum (FWHM) Gaussian kernel was implemented in TBV to allow for motion correction and spatial smoothing. The first two volumes of the functional scans were removed prior to real‐time processing. The neurofeedback target, the PCC, was defined using a 6 mm sphere over the coordinate (MNI: 0 −50 20) (Bluhm et al., 2009), and the “best voxel selection” tool in TBV was used to calculate the BOLD signal amplitude in this target area. This method identifies the 33% most active voxels (i.e., highest beta‐values) for the *view > neutral* contrast. The first two trials comprising each neurofeedback run were the *view* and *neutral* conditions to enable an initial voxel selection based on the *view > neutral* contrast, which was dynamically updated throughout the duration of training.

### 2.4 fMRI image acquisition and preprocessing

We utilized a 3 Tesla MRI Scanner (Siemens Biograph mMR, Siemens Medical Solutions, Erlangen, Germany) at the Lawson Health Research Institute with a 32‐channel head coil, where participants’ heads were stabilized during scanning. Functional whole brain images of the BOLD contrasts were acquired using a gradient echo T2*‐weighted echo‐planar‐imaging sequence (TE = 30 ms, TR = 2s, FOV = 192 × 192 mm, flip angle = 80°, inplane resolution = 3 × 3 mm). One volume consisted of 36 ascending interleaved slices tilted −20° from the AC–PC orientation. Volumes had a thickness of 3 mm and a slice gap of 1 mm. The experimental runs comprised 284 volumes each, where T1‐weighted anatomical images were obtained with a Magnetization Prepared Rapid Acquisition Gradient Echo sequence (TE = 3.03 ms, TR = 2.3s, 192 slices and FOV = 256 × 256 mm).

Functional images were preprocessed using SPM12 (Wellcome Department of Cognitive Neurology, London, UK) within MATLAB R2020a. We followed our standard preprocessing routine which included discarding the four initial volumes, slice time correction to the middle slice, reorienting to the AC‐PC axis, spatial alignment to the mean image via rigid body transformation, reslicing, and finally, coregistration of the functional mean image to the participant's anatomical image. Subsequently, we segmented the coregistered images using the “New Segment” method in SPM12. Following this step, functional images were normalized to the MNI (Montreal Neurological Institute) standard template and then smoothed using a 6 mm FWHM Gaussian kernel. Finally, we conducted additional motion correction using the Artifact Detection Tool (ART) software package (https://www.nitrc.org/projects/artifact_detect), which computes regressors to account for outlier volumes, in addition to movement regressors computed during standard realignment procedures.

### 2.5 Neurofeedback PCC downregulation analysis and state changes in emotional experience

In order to evaluate PCC downregulation (neurofeedback success), we examined the event‐related BOLD response from the PCC target sphere during the regulate and view training conditions (Nicholson et al., 2021). We also examined state changes in subjective response to traumatic/distressing stimuli over the neurofeedback training experiment, as measured by RSDI subscales (Nicholson et al., 2021). As these analyses have been previously published by our group (Nicholson et al., 2021), methodological details are included in the supplementary material (see supplementary methods s2).

### 2.6 PPI analysis

Novel analyses in the current manuscript included examining task‐dependent changes in PCC functional connectivity during neurofeedback training via PPI analyses as implemented in SPM12. A PPI analysis measures the context‐sensitive change in connectivity between one or more brain regions by comparing connectivity of the seed region during a particular experimental condition versus connectivity of the seed region during a different condition (Friston et al., 1997). In this case, we investigated the change in PCC connectivity during the view condition as compared to the regulate condition (*view > regulate*). In doing so, we were able to identify brain regions showing changes in connectivity with the PCC that are related to neurofeedback‐mediated PCC downregulation.

We extracted eigenvariates from the left and right PCC seed for each participant. Coordinates for the eigenvariates were chosen by first creating a spherical volume‐of‐interest of 6 mm centered over the PCC coordinate (MNI: 0−50 20), which was the same sphere that was used as the regulation target during our neurofeedback training paradigm. Then, for each participant, we automated selection of the nearest local activation coordinate during the contrast of *view > regulate*, while restricting the inclusion of voxels to only either the left or right hemisphere using unihemispheric gray matter masks from PickAtlas. Finally, we manually reviewed each chosen coordinate to ensure that it corresponded to the anatomical PCC region. Since we expected maximal PCC activation during the view condition, we used a contrast of the *view > regulate* conditions to extract eigenvariates for the psychological regressor.

The PPI interaction terms were created by deconvolving the BOLD signal of the physiological regressor (i.e., timeseries of the selected PCC sphere) by the hemodynamic response function and multiplying by the psychological regressor (*view > regulate*), thus creating a series of interaction terms. These interaction terms were then reconvolved with the hemodynamic response function prior to being passed on to the second‐level for within‐ and between‐group analyses. For the left and right PCC seed, across all training runs combined, we conducted a priori planned one‐ and two‐sample *t*‐tests to examine within‐ and between‐group PCC connectivity across the whole‐brain. We were also interested in assessing how PCC connectivity changed over the course of neurofeedback training and therefore conducted additional one‐sample *t*‐tests for both groups to examine PCC connectivity during training run 3 as compared to training run 1, and vice versa. Additionally, for the left and right PCC seed, we also conducted a priori planned one‐ and two‐sample *t*‐tests for the transfer run separately. Previous research by our group, including a randomized controlled trial, showed that neurofeedback is associated with normalized DMN and SN activity among individuals with PTSD (Kluetsch et al., 2014; Nicholson et al., 2020). As such, we chose to also perform a region‐of‐interest (ROI) analysis using a single mask consisting of 2 main hubs from each of the DMN (dmPFC, vmPFC) and SN (bilateral amygdala, bilateral insula). Coordinates for the midline vmPFC (*x*, *y*, *z* = −2, 36, −2) and dmPFC (*x*, *y*, *z* = 0, 34, 40) were informed by a recent meta‐analysis focusing on neurocircuitry models of PTSD (Boccia et al., 2016), as well as our previous randomized controlled trial results of EEG‐based neurofeedback in PTSD (Nicholson et al., 2020). We used PickAtlas to define 8 mm radius spheres around the vmPFC and dmPFC coordinates. The amygdala was defined using an anatomical mask from PickAtlas. Regarding the insula, we chose to examine subregions separately given observations of their unique connectivity patterns in individuals with PTSD (Nicholson et al., 2016). Insula subregions were defined using 6 mm spheres around standardized coordinates from previous anatomical and MR imaging studies (Ichesco et al., 2014; Taylor et al., 2008): bilateral anterior insula (*x*, *y*, *z* left = −32, 16, 6; right = 32, 16, 6), and bilateral posterior insula (*x*, *y*, *z* left = −39, −15, 1; right = 39, −15, 8). All coordinates are reported in MNI space. This single ROI mask was applied to all aforementioned one‐ and two‐sample *t*‐tests. Both whole‐brain and ROI analyses were corrected for multiple comparisons using a peak‐level family‐wise error (FWE) threshold at *p* < .05, *k* = 10, with an initial cluster defining threshold at *p* < .0001, *k* = 10 (Eklund et al., 2016; Roiser et al., 2016).

### 2.7 Clinical correlations

Linear regression analyses were conducted across all subjects, to evaluate correlations between clinical scores and PCC connectivity over neurofeedback training runs. Interaction term parameters were correlated with participant scores for CAPS‐5 total, DERS, CTQ, BDI, and MDI. Results were corrected for multiple comparisons using the same peak‐level FWE threshold at *p* < .05, *k* = 10, with an initial cluster defining threshold at *p* < .0001, *k* = 10 (Eklund et al., 2016; Roiser et al., 2016).

### 2.8 Regulation strategies

As previously mentioned, participants were not provided with any specific instructions regarding regulation strategies. Once participants finished their neurofeedback session and left the scanner, they were asked the following open‐ended question: “What strategies did you use?”. Participant responses to this question were then recorded, transcribed, and coded by investigators (J. M. L. and A. A. N.) utilizing thematic analytic techniques (Braun and Clarke, 2013).

## 3 RESULTS

### 3.1 PCC downregulation with neurofeedback and state changes in emotional experience

**FIGURE 2:**
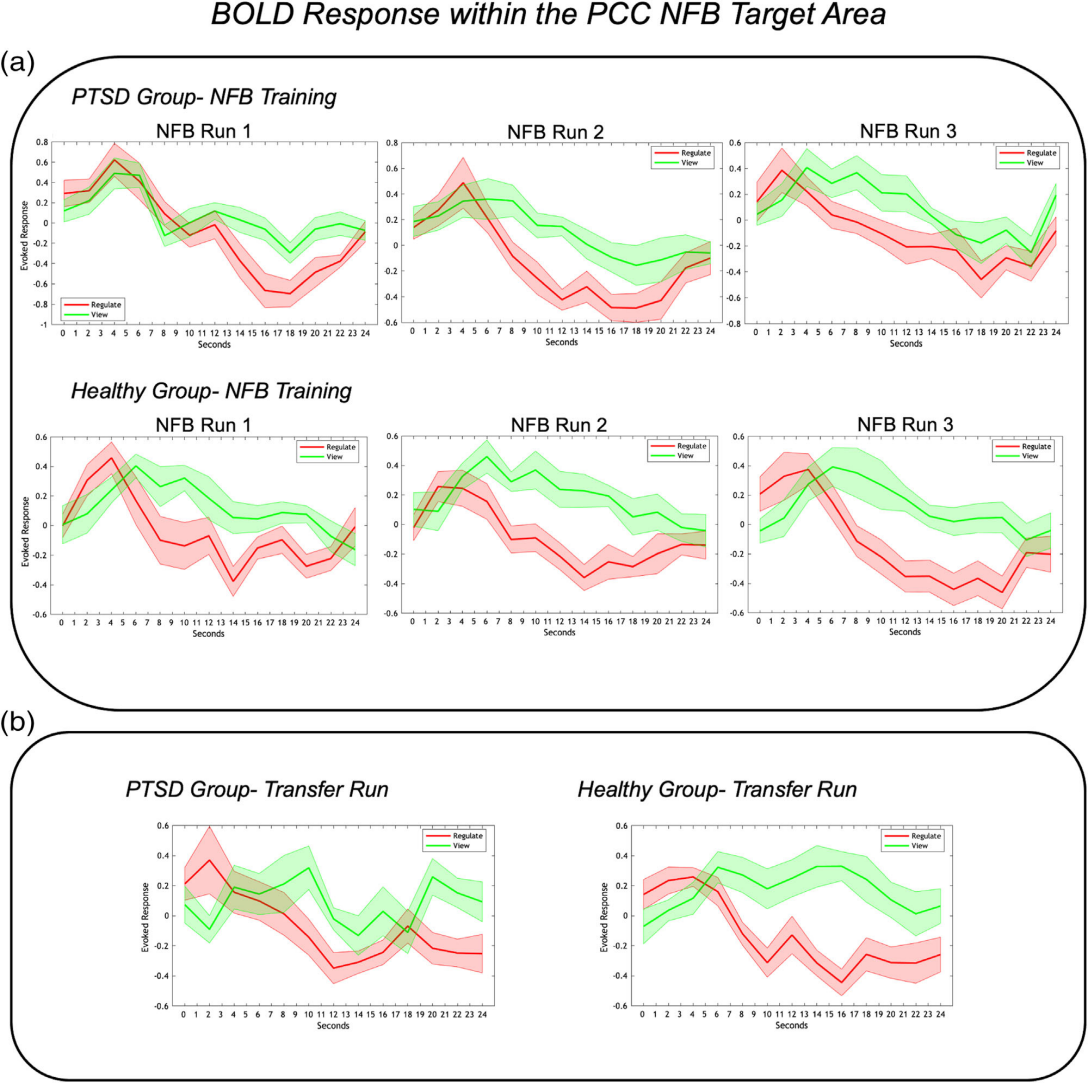
(a) Event‐related BOLD activation within the neurofeedback target area (PCC) for the PTSD and healthy control groups during the three neurofeedback training runs. The red and green lines indicate PCC activation during the *regulate* and *view* conditions, respectively. As shown, both the PTSD and healthy control groups showed significantly lower PCC activation during *regulate* as compared to *view* conditions for all three neurofeedback training runs. (b) Event‐related BOLD activation within the neurofeedback target area (PCC) for the PTSD and healthy control groups during the transfer run. As with the training runs, both groups showed significantly lower PCC activation during *regulate* as compared to *view* conditions during the transfer run. The x‐axis of the graphs indicate time over the 24 s conditions; the *y*‐axis indicates the event‐related BOLD response (peristimulus time histogram) in the neurofeedback target area (PCC). Shaded areas adjacent to the red and green lines indicate standard error of the mean. PCC = posterior cingulate cortex, NFB = Neurofeedback. Figure reproduced with permission from Nicholson et al. (2021).

**FIGURE 3:**
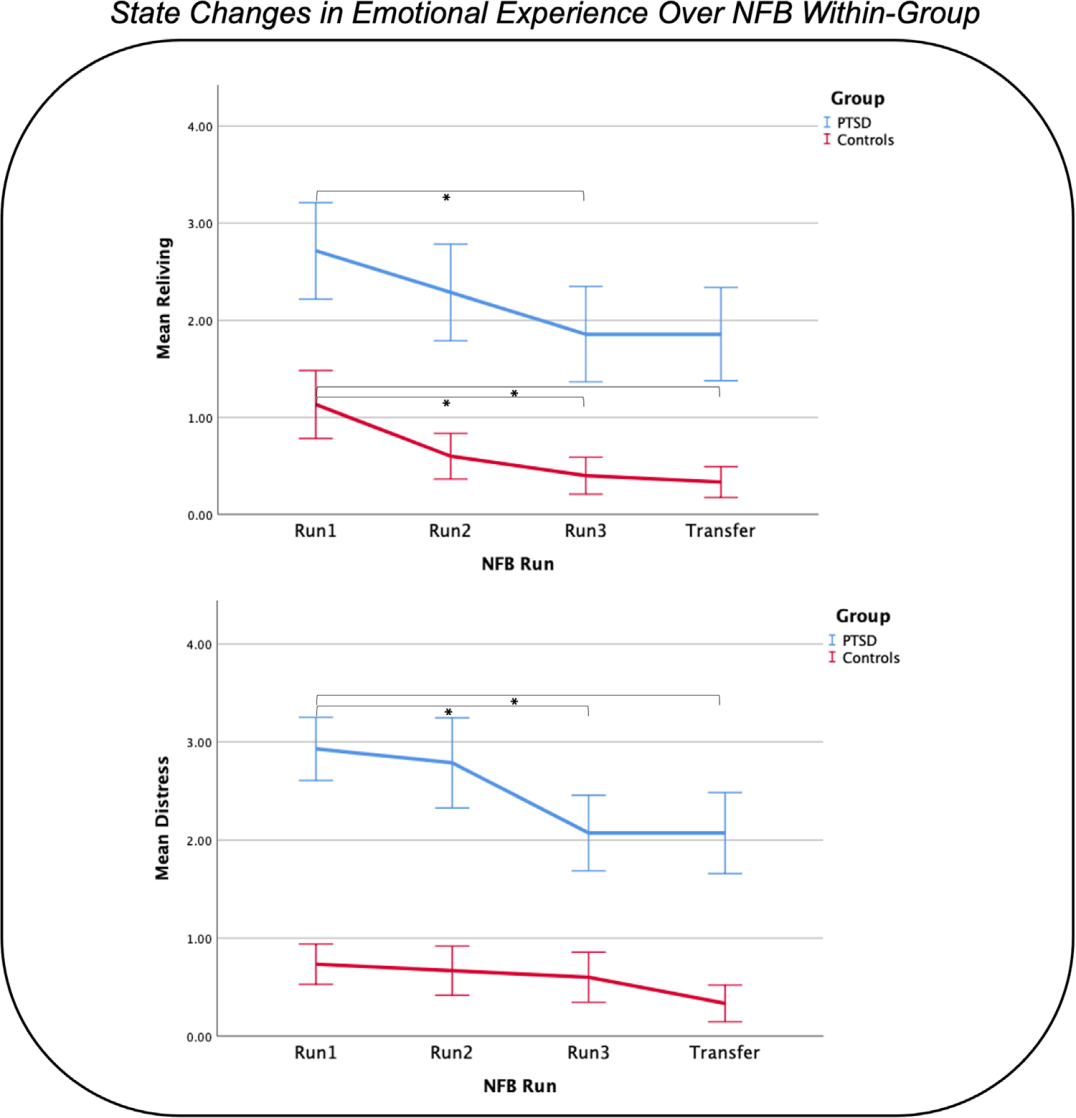
State changes in emotional experience (i.e., reliving and distress symptoms, as measured by the RSDI scale) over NFB training during a trauma/emotion provocation paradigm. Both the PTSD and healthy control groups demonstrated significant reductions in reliving symptoms. Only the PTSD group demonstrated significant reductions on distress symptoms. NFB = neurofeedback, RSDI = Response to Script Driven Imagery Scale. Figure reproduced with permission from Nicholson et al. (2021).

Individuals in both the PTSD and healthy control groups were similarly able to significantly decrease activity within their PCC (neurofeedback target area) during regulate as compared to view conditions during all three neurofeedback training runs, as well as for the transfer run (see Figure 2, as also reported elsewhere) (Nicholson et al., 2021). Indeed, there were no significant differences between the two groups when comparing the PCC BOLD responses during regulate as compared to view conditions for each of the neurofeedback training runs and transfer run (Nicholson et al., 2021). In other words, the PTSD and healthy control participants were able to regulate PCC activity with similar success (Nicholson et al., 2021). Additionally, over neurofeedback training and in response to a trauma/emotion provocation paradigm, both the PTSD and healthy control groups showed significantly reduced reliving symptoms, whereas only the PTSD group showed significant reductions in distress symptoms, as measured by the RSDI scale (see Figure 3, as also reported elsewhere) (Nicholson et al., 2021).

### 3.2 Within‐ and between‐group PPI results

#### 3.2.1 Neurofeedback training runs

**FIGURE 4:**
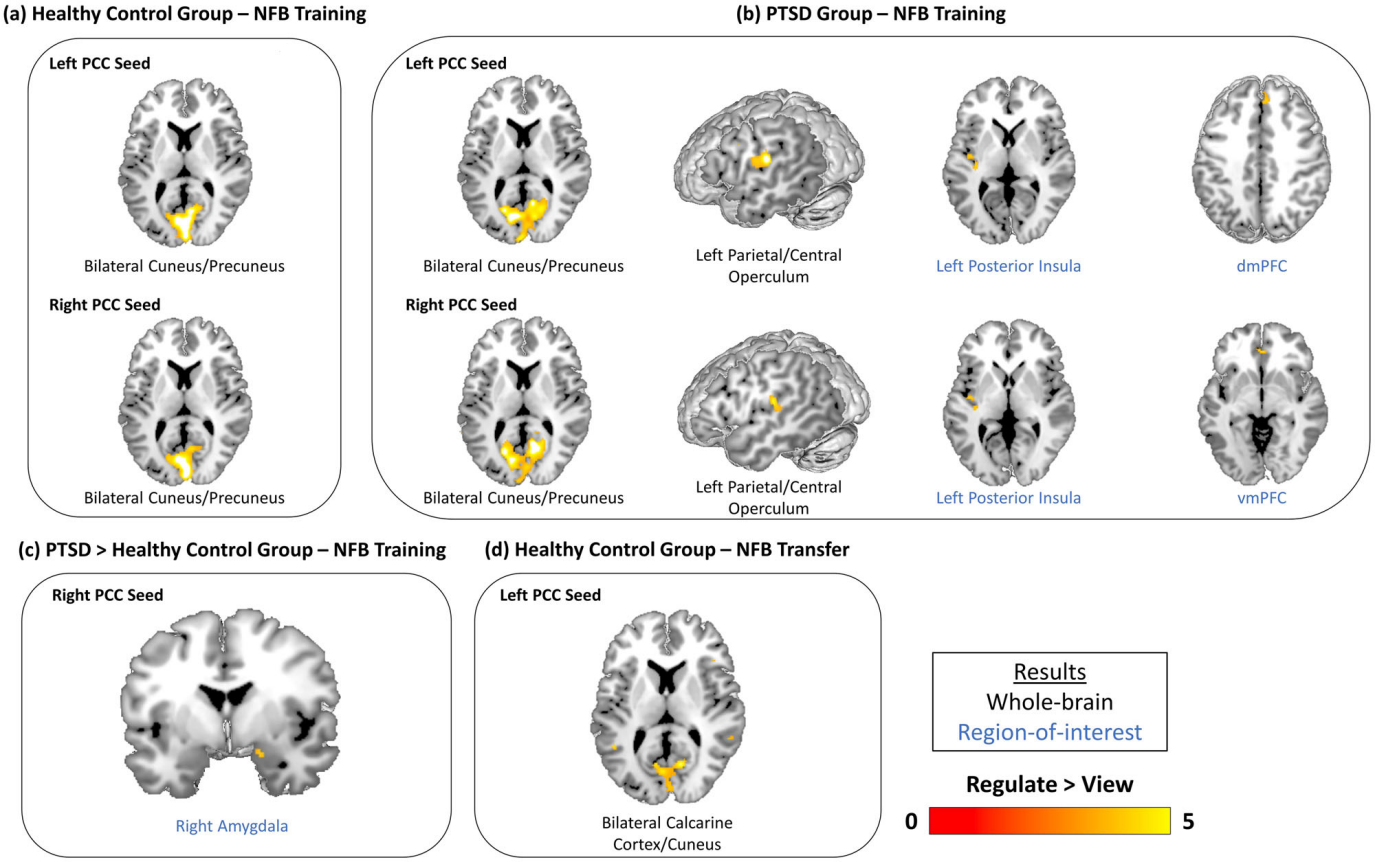
Within‐ and between‐group differences in functional connectivity of the PCC during the 3 neurofeedback training runs and the transfer run. Results show brain areas that were found to display increased functional connectivity with the PPI seed region (left or right PCC) during regulate as compared to view conditions. (a) The within‐healthy control group comparison revealed left and right PCC connectivity with the bilateral cuneus/precuneus during regulate as compared to view conditions for the neurofeedback training runs. (b) The within‐PTSD group comparison revealed left and right PCC connectivity with the bilateral cuneus/precuneus, left parietal/central operculum, and left posterior insula during regulate as compared to view conditions for the neurofeedback training runs. Additionally, left PCC‐dmPFC and right PCC‐vmPFC connectivity was also observed during regulate as compared to view conditions for the neurofeedback training runs. (c) The between‐group comparison revealed that the PTSD group displayed increased right PCC‐right amygdala connectivity relative to the healthy control group during regulate as compared to view conditions for the neurofeedback training runs. (d) The within‐healthy control group comparison revealed left PCC connectivity with the bilateral calcarine cortex/cuneus during regulate as compared to view conditions for the neurofeedback transfer run. All results are evaluated at the FWE‐peak corrected threshold for multiple comparisons (*p* < .05, *k* = 10). NFB = neurofeedback, PCC = posterior cingulate cortex, dmPFC = dorsomedial prefrontal cortex, vmPFC = ventromedial prefrontal cortex.

Both the PTSD and healthy control groups demonstrated significant whole‐brain PCC connectivity with the bilateral precuneus/cuneus during regulate as compared to view neurofeedback training conditions (see Table 2 and Figures 4a and b). However, only the PTSD group showed significant whole‐brain PCC connectivity with the left parietal/central operculum during regulate as compared to view conditions during neurofeedback training (see Table 2 and Figure 4b).

**TABLE 2:** Within‐ and between‐group differences in functional connectivity of the PCC during neurofeedback training runs

						MNI coordinate					
Comparison	Contrast	PCC seed	Brain region	*H*	*k*	*x*	*y*	*z*	*t*‐Stat.	*z*‐Score	*p*‐FWE peak
**Whole‐brain**											
**Within‐group**											
Healthy controls	View > Reg	Left	ns								
	View > Reg	Right	ns								
	Reg > View	Left	Precuneus/cuneus		3241	4	−82	20	6.57	6.17	<.001
	Reg > View	Right	Precuneus/cuneus		2877	4	−82	18	6.32	5.96	<.001
PTSD	View > Reg	Left	ns								
	View > Reg	Right	ns								
	Reg > View	Left	Parietal/central operculum	L	227	−52	−26	16	5.94	5.64	.001
			Precuneus/cuneus		2862	−10	−72	12	5.84	5.56	.002
	Reg > View	Right	Precuneus/cuneus		3442	10	−58	10	5.93	5.63	.001
			Parietal/central operculum	L	435	−52	−26	16	5.29	5.07	.019
**Between‐group**											
PTSD > Healthy controls	View > Reg	Left	ns								
	View > Reg	Right	ns								
	Reg > View	Left	ns								
	Reg > View	Right	ns								
**ROIs**											
**Within‐group**											
Healthy controls	View > Reg	Left	ns								
	View > Reg	Right	ns								
	Reg > View	Left	ns								
	Reg > View	Right	ns								
PTSD	View > Reg	Left	ns								
	View > Reg	Right	ns								
	Reg > View	Left	dmPFC		20	4	38	38	3.99	3.89	.040
			Posterior insula	L	25	−38	−12	4	3.97	3.87	.043
	Reg > View	Right	vmPFC		42	0	38	−4	4.53	4.39	.006
			Posterior insula	L	18	−38	−12	4	3.97	3.87	.042
**Between‐group**											
PTSD > Healthy controls	View > Reg	Left	ns								
	View > Reg	Right	ns								
	Reg > View	Left	ns								
	Reg > View	Right	Amygdala	R	23	16	−6	−14	4.05	3.94	.033

*Note*: Within‐ and between‐group differences in functional connectivity of the PCC (left and right seeds) during the neurofeedback training runs. Reported results for whole‐brain and ROI analyses are at a significance threshold of *p*‐FWE peak‐level < .05, k = 10. The comparison column lists each group comparison, and the contrast column indicates the direction in which the regulate and view conditions are being contrasted (i.e., view > regulate or regulate > view). The brain region, hemisphere of the region (*H*), cluster size (*k*), MNI coordinates (*x*, *y*, *z*), *t*‐statistic (*t*‐stat.), *z*‐Score, and significance (*p*‐FWE peak) of each significant peak result are included as columns. PCC = posterior cingulate cortex, FWE = family‐wise error, PTSD = posttraumatic stress disorder, Reg = regulate, ns = nonsignificant, L = left, R = right, dmPFC = dorsomedial prefrontal cortex, vmPFC = ventromedial prefrontal cortex.

Interestingly, the ROI analyses revealed that only the PTSD group displayed significant PCC functional connectivity to the DMN and SN during regulate as compared to view neurofeedback training conditions. Both the left and right PCC seeds were found to yield significant functional connectivity with the left posterior insula during regulate as compared to view conditions (see Table 2 and Figure 4b). Additionally, both PCC seeds were also found to yield significant PCC connectivity with PFC regions—the left PCC to the dorsomedial prefrontal cortex (dmPFC) and the right PCC to the ventromedial prefrontal cortex (vmPFC)—during regulate as compared to view conditions (see Table 2 and Figure 4b). Furthermore, in the between‐group ROI analysis, the right PCC was found to display significantly greater connectivity with the right amygdala for the PTSD group as compared to the control group, during regulate as compared to view neurofeedback training conditions (see Table 2 and Figure 4c). Lastly, we did not detect any significant changes in PCC connectivity over the course of neurofeedback training (i.e., run 3 as compared to run 1, and vice versa) during regulate as compared to view, and vice versa.

#### 3.2.2 Transfer run

Within‐group whole‐brain analyses of PCC functional connectivity during the transfer run revealed that the healthy control group showed left PCC connectivity with the bilateral calcarine cortex/cuneus during regulate as compared to view conditions (see Table 3 and Figure 4d). For both whole‐brain and ROI analyses, there were nonsignificant differences in PCC connectivity within the PTSD group and when comparing between the PTSD and healthy control groups.

**TABLE 3:** Within‐ and between‐group differences in functional connectivity of the PCC during the neurofeedback transfer run

					MNI coordinate					
Comparison	Contrast	PCC seed	Brain region	*k*	*x*	*y*	*z*	*t*‐Stat.	*z*‐Score	*p*‐FWE peak
**Whole‐brain**										
** Within‐group**										
Healthy controls	View > Reg	Left	ns							
	View > Reg	Right	ns							
	Reg > View	Left	Calcarine cortex/cuneus	1272	14	−64	6	5.86	5.13	.019
	Reg > View	Right	ns							
PTSD	View > Reg	Left	ns							
	View > Reg	Right	ns							
	Reg > View	Left	ns							
	Reg > View	Right	ns							
**Between‐group**										
PTSD > Healthy controls	View > Reg	Left	ns							
	View > Reg	Right	ns							
	Reg > View	Left	ns							
	Reg > View	Right	ns							
**ROIs**										
**Within‐group**										
Healthy controls	View > Reg	Left	ns							
	View > Reg	Right	ns							
	Reg > View	Left	ns							
	Reg > View	Right	ns							
PTSD	View > Reg	Left	ns							
	View > Reg	Right	ns							
	Reg > View	Left	ns							
	Reg > View	Right	ns							
**Between‐group**										
PTSD > Healthy controls	View > Reg	Left	ns							
	View > Reg	Right	ns							
	Reg > View	Left	ns							
	Reg > View	Right	ns							

*Note*: Within‐ and between‐group differences in functional connectivity of the PCC (left and right seeds) during the neurofeedback transfer run. Reported results for whole‐brain and ROI analyses are at a significance threshold of *p*‐FWE peak‐level < .05, *k* = 10. The comparison column lists each group comparison, and the contrast column indicates the direction in which the regulate and view conditions are being contrasted (i.e., view > regulate or regulate > view). The brain region, cluster size (*k*), MNI coordinates (*x*, *y*, *z*), *t*‐statistic (*t*‐stat.), *z*‐Score, and significance (*p*‐FWE peak) of each significant peak result are included as columns. PCC = posterior cingulate cortex, FWE = family‐wise error, PTSD = posttraumatic stress disorder, Reg = regulate, ns = nonsignificant.

### 3.3 Clinical correlations

**FIGURE 5:**
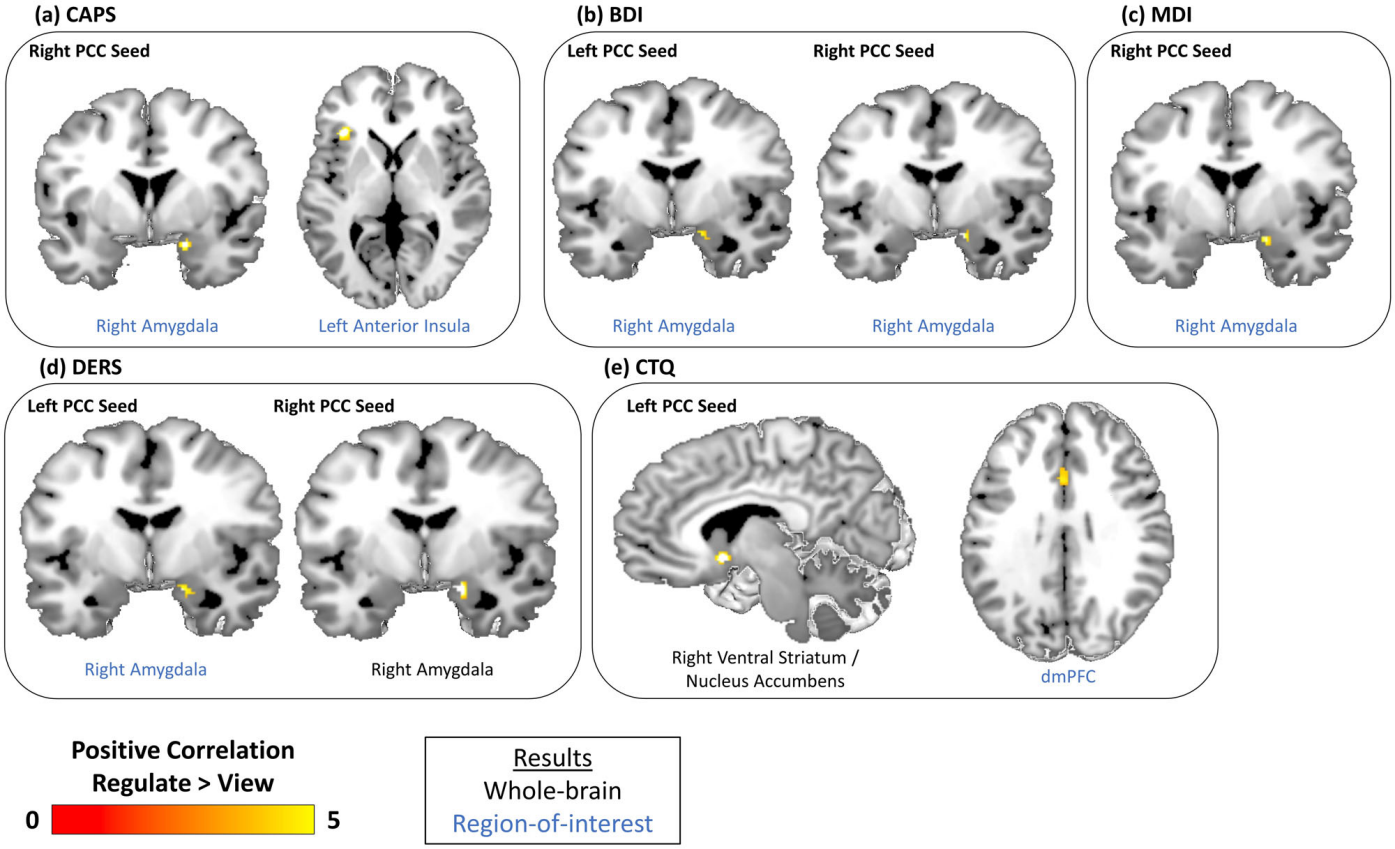
Clinical symptom correlation results across all participants over 3 combined neurofeedback training runs. Results indicate brain areas whose connectivity with the PPI seed region—the left or right PCC—are correlated with participant scores on clinical measures during regulate as compared to view conditions. (a) CAPS scores are positively correlated with connectivity between the right PCC and both the right amygdala and left anterior insula. (b) Connectivity between both PCC seeds and the right amygdala are positively correlated with BDI scores. (c) MDI scores are positively correlated with right PCC‐right amygdala connectivity. (d) Connectivity between both PCC seeds and the right amygdala are positively correlated with DERS scores. (e) CTQ scores are positively correlated with connectivity between the left PCC and both the right ventral striatum/nucleus accumbens and the dmPFC. All results are evaluated at the FWE‐peak corrected threshold for multiple comparisons (*p* < .05, *k* = 10). NFB = neurofeedback, PCC = posterior cingulate cortex, dmPFC = dorsomedial prefrontal cortex, CAPS = Clinician‐administered PTSD Scale, BDI = Beck's Depression Inventory, MDI = Multiscale Dissociation Inventory, DERS = Difficulties in Emotion Regulation Scale, CTQ = Childhood Trauma Questionnaire.

We found several significant results when conducting linear regression analyses between PCC functional connectivity and psychiatric clinical symptoms across all participants during neurofeedback training runs (see Table 4 and Figure 5). In the whole‐brain analysis, a positive correlation was detected between CTQ scores and left PCC‐right ventral striatum/nucleus accumbens (VS/NAcc) connectivity during regulate as compared to view neurofeedback training conditions. There was also a positive correlation detected between DERS and right PCC‐right amygdala connectivity during regulate as compared to view neurofeedback training conditions.

**TABLE 4:** Clinical symptom correlations with PCC connectivity during neurofeedback training runs

						MNI coordinate					
Measure	Direction	PCC seed	Brain region	*H*	*k*	*x*	*y*	*z*	*t*‐Stat.	*z*‐Score	*p*‐FWE peak
**Reg > View (Whole‐brain)**											
CTQ	+	Left	Ventral striatum/nucleus accumbens	R	46	6	8	−6	7.35	5.40	.006
DERS	+	Right	Amygdala	R	71	16	−6	−12	6.59	5.04	.039
**Reg > View (ROIs)**	+										
BDI	+	Left	Amygdala	R	14	16	−6	−14	4.77	4.02	.032
	+	Right	Amygdala	R	22	16	−6	−12	5.28	4.34	.009
CAPS‐5 total	+	Right	Amygdala	R	34	20	0	−16	5.25	4.32	.009
	+	Right	Anterior insula	L	28	−32	22	6	4.79	4.04	.032
CTQ	+	Left	dmPFC		37	4	40	38	4.65	3.95	.042
DERS	+	Left	Amygdala	R	28	16	−6	−14	5.05	4.20	.016
	+	Right	Amygdala	R	47	16	−6	−12	6.59	5.04	<.001
MDI	+	Right	Amygdala	R	24	16	−6	−12	5.86	4.66	.002

*Note*: Clinical correlations from the linear regression analyses between clinical scores and PCC functional connectivity during the neurofeedback training runs. The linear regression analyses were conducted across all study participants. Reported results for whole‐brain and ROI analyses are at a significance threshold of *p*‐FWE peak‐level < .05, *k* = 10. The measure column lists the specific clinical inventory or questionnaire administered. The direction of correlation (±), PCC seed, brain region, hemisphere of the region (*H*), cluster size (*k*), MNI coordinates (*x*, *y*, *z*), *t*‐statistic (*t*‐stat.), *z*‐Score, and significance (*p*‐FWE peak) for each significant peak result are included as columns. PPI = psychophysiological interaction, PCC = posterior cingulate cortex, H = hemisphere, FWE = family‐wise error, L = left, R = right, dmPFC = dorsomedial prefrontal cortex, CTQ = Childhood Trauma Questionnaire, DERS = Difficulties in Emotion Regulation Scale, BDI—Beck's Depression Inventory, CAPS = Clinician‐Administered PTSD Scale, MDI = Multiscale Dissociation Inventory.

In the ROI analysis, positive correlations were found between BDI, CAPS, DERS, and MDI scores and right PCC‐right amygdala connectivity during regulate as compared to view neurofeedback training conditions. Additionally, positive correlations were found for both BDI and DERS scores with left PCC‐right amygdala connectivity during regulate as compared to view neurofeedback training conditions. Moreover, a positive correlation was found for CAPS scores and right PCC‐left anterior insula connectivity and for CTQ scores and left PCC‐dmPFC connectivity during regulate as compared to view conditions.

### 3.4 Regulation strategies

In the present study, participants were not provided with any instructions regarding strategies for neurofeedback regulation. Interestingly, we observed the recurrence of several consistent themes across the self‐reported regulation strategies utilized by participants. Indeed, the most commonly reported strategy was relating to the monitoring and/or alteration of the breath (e.g., deep breathing, breath as an anchor, etc.). Other common strategies reported by participants include self‐talk, and the use of visual imagery. Notably, the majority of participants reported utilizing two or more regulatory strategies throughout the session.

## 4 DISCUSSION

In our previously published analysis, we reported that a single session of successful PCC‐downregulation with rt‐fMRI‐NFB facilitated emotion regulation among individuals with PTSD, where psychological symptoms of reliving and distress were reduced during a trauma/emotion provocation paradigm (Nicholson et al., 2021). Indeed, this corresponded to unique patterns of brain activation among individuals with PTSD and healthy controls during neurofeedback training (Nicholson et al., 2021). In the present analysis, comparing functional connectivity of the PCC during regulate as compared to view conditions permits further insight into neurobiological mechanisms that may underlie neurofeedback‐mediated PCC downregulation and allow us to assess whether PTSD and healthy control participants recruit different neural mechanisms to facilitate effective regulation. Here, within‐ and between‐group PCC functional connectivity results suggest that downregulating PCC activity with rt‐fMRI‐NFB may recalibrate PTSD‐associated alterations in SN and DMN connectivity, particularly with regard to a priori specified limbic (i.e., amygdala) and cortical regions (i.e., insular and prefrontal cortices) that are central to emotion generation, processing, and regulation. These results, if replicated, may call for an expansion of neural mechanistic models to include the involvement of the PCC in facilitating emotion regulation among individuals with PTSD.

During neurofeedback training, the PTSD and healthy control groups showed both similarities and differences regarding PCC functional connectivity to major hubs within the SN (i.e., amygdala and posterior insula) and DMN (i.e., dmPFC, vmPFC, and precuneus). Specifically, in the neurofeedback training runs, while both the PTSD and healthy control groups showed PCC connectivity with the posterior DMN (i.e., precuneus/cuneus) during regulate as compared to view conditions, only the PTSD group showed PCC connectivity with the SN (i.e., posterior insula) and the anterior DMN (i.e., dmPFC, vmPFC). Furthermore, as compared to healthy controls, the PTSD group was shown to have significantly greater PCC functional connectivity with the amygdala during regulate as compared to view conditions. Additionally, during neurofeedback training runs, higher psychiatric symptom (i.e., CAPS total, CTQ, BDI, MDI, DERS) scores were positively correlated with connectivity between the PCC and hubs within both the DMN (i.e., dmPFC) and SN (i.e., amygdala, anterior insula, VS/NAcc). These linear regression results may indicate that individuals with more severe symptoms maintain a greater need for network recalibration in comparison with those with milder symptoms. Importantly, observed patterns of DMN and SN connectivity during neurofeedback, as well as correlations with psychiatric symptoms, are concordant with connectivity results from both our pilot study (Kluetsch et al., 2014) and randomized controlled trial (Nicholson et al., 2020) of alpha‐rhythm EEG neurofeedback.

### 4.1 Default mode network

As previously discussed, individuals with PTSD have been shown to demonstrate decreased functional coupling within the DMN at rest (Bluhm et al., 2009; Qin et al., 2012; Koch et al., 2016; Sripada et al., 2012), whereas increased functional coupling has been found during script‐driven imagery and trauma‐related processing (Fenster et al., 2018; Frewen et al., 2011; Hopper et al., 2007; Liberzon & Abelson, 2016; Mickleborough et al., 2011; Ramage et al., 2013; Thome et al., 2020). Importantly, among individuals with PTSD, extensive research has demonstrated PCC hyperactivity during the reliving and reexperiencing of trauma memories (Fenster et al., 2018; Frewen et al., 2011; Hopper et al., 2007; Liberzon & Abelson, 2016; Mickleborough et al., 2011; Ramage et al., 2013; Thome et al., 2020). Hence, we predicted that downregulating the PCC during symptom provocation via rt‐fMRI‐NFB may help to recalibrate connectivity within the DMN among individuals with PTSD.

During neurofeedback training, we observed both commonalities and differences between the PTSD and healthy control groups with respect to PCC‐DMN connectivity. Both groups were found to exhibit PCC‐precuneus/cuneus connectivity during regulate as compared to view conditions. Conversely, there was unique within‐PTSD group connectivity between the PCC and both the dmPFC and vmPFC during regulate as compared to view training conditions. These results are concordant with previous PTSD neuroimaging studies which have found distinct patterns of connectivity within different communities of the DMN, namely that connectivity within the posterior DMN may be spared relative to decreased connectivity within the anterior DMN among individuals with PTSD (Akiki et al., 2018; Holmes et al., 2018; Kennis et al., 2016; Shang et al., 2014). Hence, the observed group similarity in posterior DMN connectivity during PCC downregulation appears to further reinforce the notion that connectivity within the posterior DMN is, relative to anterior DMN connectivity, unperturbed in PTSD and thus its functionality may remain intact. At the same time, unique within‐PTSD group PCC‐anterior DMN connectivity suggests that PCC‐targeted rt‐fMRI‐NFB may help to normalize connectivity patterns within the anterior DMN, as well as integrate functionally segregated anterior and posterior DMN communities. This may represent a neurofeedback‐mediated recalibration of PTSD‐associated alterations in DMN connectivity. These findings are also in alignment with results from our group's randomized controlled trial of EEG neurofeedback, wherein participants trained to downregulate alpha amplitude using real‐time EEG feedback signals from the PCC/posterior DMN (Nicholson et al., 2020). In this study, decreased PTSD severity scores coincided with increased resting‐state connectivity between the DMN and the dmPFC after NFB training, which may also reflect a recalibration of aberrant connectivity patterns associated with PTSD.

Although dorsomedial and ventromedial DMN communities have differences with regard to cytoarchitecture and anatomical connectivity, as well as some degree of functional segregation (Raichle, 2015), both are consistently implicated in PTSD psychopathology (Bluhm et al., 2009; Daniels et al., 2010; Lanius et al., 2010). Indeed, the dmPFC and vmPFC have both been repeatedly demonstrated to be hypoactive during the reliving and reexperiencing of traumatic experiences, as well as the processing of fearful facial expressions, among individuals with PTSD (Killgore et al., 2014; Lanius et al., 2007; Peres et al., 2011; Shin et al., 2005; Williams et al., 2006). Interestingly, the dmPFC was also identified in our linear regression analysis, where there was a positive correlation between PCC‐dmPFC connectivity and CTQ scores during regulate as compared to view conditions. Severe and/or prolonged childhood trauma has been shown to have a destabilizing impact on the developmental differentiation of the DMN, particularly with regard to integration between the anterior and posterior DMN subsystems (Bluhm et al., 2009; Daniels et al., 2011). As such, the observed correlation may indicate that individuals experiencing severe developmental traumas have a greater need to functionally couple anterior and posterior DMN activity during neurofeedback. Taken together, functional connectivity between the PCC/posterior DMN and the anterior DMN, including both the vmPFC and dmPFC, supports the notion that downregulating the PCC via rt‐fMRI‐NFB may recalibrate altered DMN connectivity patterns that are associated with PTSD psychopathology (Akiki et al., 2017; Bluhm et al., 2009; Daniels et al., 2010; Fenster et al., 2018; Lanius et al., 2015; Tursich et al., 2015).

### 4.2 Salience network

Altered connectivity within the SN among individuals with PTSD has been well established by the existing knowledge base (Akiki et al., 2017; Fenster et al., 2018; Lanius et al., 2015; Vanasse et al., 2019; Wang et al., 2016) and has been associated with specific PTSD symptoms of hyperarousal, hypervigilance, avoidance, and altered interoception (Akiki et al., 2017; Koch et al., 2016; McCurry et al., 2020; Nicholson et al., 2020; Rabinak et al., 2011; Sripada et al., 2012; Tursich et al., 2015; Yehuda et al., 2015). Indeed, there is evidence to suggest that SN activity predominates resting‐state processing in individuals with PTSD as opposed to typical resting‐state DMN activity observed in healthy controls (Harricharan et al., 2020; Lanius et al., 2015; Nicholson et al., 2020; Nicholson et al., 2016; Sripada et al., 2012). During neurofeedback training, we observed unique patterns of PCC‐SN connectivity among PTSD participants. The within‐PTSD group comparison revealed significant PCC‐left posterior insula connectivity during regulate as compared to view conditions. Additionally, the linear regression analysis revealed a positive correlation between right PCC‐left anterior insula connectivity and CAPS scores during regulate compared to view. The insula, a core cortical region of the SN, is integral to the processing of emotional states and can be parcellated into functionally differentiated anterior, mid‐, and posterior subregions (Craig, 2009, Chang et al., 2013; Couto et al., 2013; Craig, 2002). Interestingly, a posterior‐to‐mid‐to‐anterior integration of interoceptive information (i.e., signals related to the internal state of the body) within the insular cortex has been proposed and substantiated by functional neuroimaging research (Craig, 2009, Cauda et al., 2012; Chang et al., 2013; Couto et al., 2013; Craig, 2002; Simmons et al., 2013). According to this model, primary interoceptive signals are first represented in the posterior insula and integrated with external (environmental) and sensory information along the processing pathway (Craig, 2009, Cauda et al., 2012; Chang et al., 2013; Couto et al., 2013; Craig, 2002; Simmons et al., 2013). Subsequently, the anterior insula is critically involved in attributing salience and facilitating higher‐order emotional and perceptive processing via interactions with frontal brain regions, including particularly strong functional interactions with the anterior cingulate cortex (Craig, 2009, Beissner et al., 2013; Cauda et al., 2011; Uddin, 2015). In PTSD, dysregulation within the insular cortex—namely, posterior insula hypoactivity and anterior insula hyperactivity (Harricharan et al., 2020; Koch et al., 2016; Patel et al., 2012; Wang et al., 2016)—may reflect a disrupted balance between interoceptive and exteroceptive processing, as well as maladaptive emotional reactivity to trauma‐related stimuli and altered bodily self‐consciousness. Indeed, extensive research has linked altered connectivity within subregions of the insular cortex to particular PTSD symptoms (Akiki et al., 2017; Koch et al., 2016; McCurry et al., 2020; Nicholson et al., 2020; Rabinak et al., 2011; Sripada et al., 2012; Tursich et al., 2015; Yehuda et al., 2015). In one study, Tursich et al. (2015) analyzed network connectivity via an independent component analysis and found decreased left posterior insula integration within the SN as a function of hyperarousal symptoms, along with opposing trend‐level SN associations of overlapping clusters, including the anterior insula, as a function of reexperiencing (increased connectivity) and avoidance/numbing (decreased connectivity). Additionally, our observation of PCC connectivity with only the left (as opposed to bilateral or only the right) posterior insula may be consistent with research suggesting that affective processing may entail critical involvement of the left posterior insula in particular (Duerden et al., 2013), although future research in this area is warranted. Taken together, regulating PCC activity via neurofeedback may be an effective approach in recalibrating PTSD‐associated alterations within the insular cortex.

With regard to the linear regression analysis, another critical positive correlation was found between left PCC‐right ventral striatum/nucleus accumbens (VS/NAcc) connectivity and CTQ scores during regulate compared with view neurofeedback training conditions. Although not specified as a ROI ahead of the present analysis, the VS/NAcc is a key hub of the SN and is critically involved in reward‐based brain networks (Menon, 2011; Peters et al., 2016; Seeley et al., 2007). Significantly, childhood adversity and/or trauma has been shown to alter neural connectivity, including in the VS/NAcc, pertaining to an individual's *seeking system* (Birnie et al., 2020; McLaughlin et al., 2019), a system whose activation determines one's attitude and/or disposition towards their environment (Alcaro & Panksepp, 2011). Indeed, such alterations in neural connectivity may explain the occurrence of negatively valenced *seeking behaviors* that are commonly observed among individuals with PTSD, including elevated risk‐taking (e.g., thrill seeking, aggression) (Contractor et al., 2017; Seidemann et al., 2021; Strom et al., 2012; Weiss et al., 2013), impulsivity (Kim & Choi, 2020; Mahoney et al., 2020; Weiss et al., 2012), and substance use (Mahoney et al., 2020; Michaels et al., 2021; Norman et al., 2018). Interestingly, convergent evidence has also indicated a modulatory effect of the VS/NAcc on the insular cortex by providing incentive signals as part of the integration of salience from sensory stimuli (Leong et al., 2016; Leong et al., 2021; Menon & Levitin, 2005; Perry et al., 2019).

Most importantly, however, results from the between‐group comparison further supported the notion that regulating the PCC via neurofeedback may recalibrate SN connectivity. Specifically, we observed greater PCC‐right amygdala connectivity for the PTSD as compared to the healthy control group during regulate as compared to view neurofeedback training conditions. Hyperactivity within the amygdala—a brain region associated with emotion generation and processing—has been repeatedly implicated in PTSD psychopathology (Aghajani et al., 2016; Birn et al., 2014; Etkin et al., 2011; Fenster et al., 2018; Fitzgerald et al., 2018; Koch et al., 2016; Lanius et al., 2015; Lanius et al., 2010; Mickleborough et al., 2011; Patel et al., 2012; Yehuda et al., 2015). Moreover, alterations in amygdala connectivity are involved in multiple PTSD symptom domains, including avoidance, reexperiencing, and altered perception of valence (Fenster et al., 2018), in addition to its well‐established association with hyperarousal and hyperreactivity symptoms. As such, downregulating the amygdala has been the most common target of previous rt‐fMRI‐NFB studies in PTSD (Chiba et al., 2019; Gerin et al., 2016; Misaki et al., 2019; Misaki et al., 2018; Nicholson et al., 2018; Nicholson et al., 2016; Zotev et al., 2018). Notably, in a previous EEG‐based neurofeedback study by our group, we demonstrated a shift in amygdala connectivity from brain areas involved in defensive, emotional, and fear processing/memory retrieval (i.e., PAG and hippocampus) toward prefrontal areas that facilitate emotion regulation and executive functioning (i.e., vmPFC) (Nicholson et al., 2016). Indeed, given the central involvement of the amygdala in PTSD psychopathology, and in emotion generation/processing generally, observed PCC‐amygdala functional connectivity is of particular importance. Furthermore, the linear regression analysis revealed positive correlations between right PCC‐right amygdala connectivity and CAPS, BDI, MDI, and DERS scores, as well as between left PCC‐right amygdala connectivity and BDI and DERS scores during regulate as compared to view conditions. These positive correlations may reflect the fact that individuals with more severe psychiatric symptoms (i.e., CAPS total, CTQ, BDI, MDI, DERS) require greater recalibration of amygdala connectivity during neurofeedback‐mediated PCC downregulation, as compared to individuals with less severe symptoms. Taken together, these results suggest that PCC‐targeted neurofeedback may be effective in recalibrating altered SN (i.e., amygdala and insula) connectivity to restore emotion regulation processes in PTSD. Indeed, this notion is further supported by our previously published PCC neurofeedback analysis which revealed improved emotion regulation (i.e., reduced distress and reliving symptoms) among patients with PTSD during exposure to trauma‐related stimuli (Nicholson et al., 2021).

### 4.3 Expanding neurobiological models of emotion regulation in PTSD

The manifestation and maintenance of PTSD symptoms have been linked to impaired emotion regulation capacities among individuals with this psychiatric disorder. As such, understanding the neural mechanisms underlying emotion regulation has been a priority for PTSD researchers and significant progress has been made. In PTSD, hypoactivity within the prefrontal cortex has been extensively reported and has been linked to impairments in emotion regulation (Andrewes & Jenkins, 2019; Fenster et al., 2018; Koenigs & Grafman, 2009; Lobo et al., 2011; Sripada et al., 2012). Indeed, such impairments among individuals with PTSD are commonly understood to involve reduced inhibition from prefrontal cognitive control systems on the subcortical limbic regions (i.e., the amygdala) that generate emotion (Comte et al., 2016; Gross, 1998; Ochsner & Gross, 2005; Ochsner et al., 2012). More recently, neurobiological evidence indicates that the DMN and the PCC are intimately linked with the limbic system and emotion generation areas and, as such, are critical targets for enabling healthy emotion regulation in the context of PTSD.

For example, in a recent meta‐analysis, functional deactivations in the PCC were found to constitute the specific neural substrate underlying emotional acceptance ‐ an emotion regulation strategy promoted by a wide variety of psychotherapeutic practices (Messina et al., 2021). Interestingly, in a rt‐fMRI‐NFB study targeting PCC downregulation, mindfulness‐based meditation was found to be associated with deactivation of the PCC (Garrison et al., 2013a; Garrison et al., 2013b). In a different study, increased PCC‐ACC and PCC‐anterior insula functional connectivity were associated with greater decentering, a specific metacognitive strategy for emotion regulation, among individuals with co‐occurring anxiety and depressive disorders (Fresco et al., 2017). With regard to PTSD specifically, a recent study reported decreases in both PCC and amygdala activation in conjunction with reduced PTSD symptoms among adolescents following trauma‐focused cognitive behavioral therapy (Garrett et al., 2019). Additionally, the strength of PCC‐amygdala connectivity has been demonstrated to have predictive value both in terms of positively correlating to PTSD symptom severity (Lanius et al., 2010; Zhou et al., 2012) and negatively correlating to treatment response among patients with PTSD post‐intervention (Sheynin et al., 2020). Furthermore, neurofeedback‐mediated regulation of the PCC has previously been shown to facilitate improvements in emotion regulation during a single‐session (Kluetsch et al., 2014; Nicholson et al., 2021) and reduce PTSD symptoms in a 20‐week randomized controlled trial (Nicholson et al., 2020). Taking these findings into consideration, downregulating the PCC may represent a fruitful approach for recalibrating aberrant limbic connectivity and facilitating emotion regulation in the context of PTSD.

Indeed, in the current study we observed PCC functional connectivity to subcortical limbic regions (i.e., amygdala) and limbic‐related cortices (i.e., posterior insula, prefrontal cortex) that was unique to PTSD participants during rt‐fMRI‐NFB training. The observed functional connectivity results suggest that by regaining control over PCC activity, individuals with PTSD may be able to recalibrate aberrant connectivity within their subcortical limbic system and related cortical brain structures/regions. In doing so, they may be better able to regulate negative emotions that are central to PTSD symptomatology as evidenced by the observed decreases in reliving and distress symptoms. Taken together, existing neural mechanistic models of emotion regulation may benefit from expanding to include the involvement of the PCC/DMN in facilitating emotion regulation among individuals with PTSD.

#### 4.3.1 Future directions and limitations

First, despite concordance with previous neurofeedback investigations in PTSD, we are unable to definitively conclude that these study findings are specifically attributable to PCC‐targeted neurofeedback (i.e., neurophysiological specificity), as we did not include a sham‐control condition. Second, we did not collect data on, and were thus unable to control for socio‐economic status (including educational attainment) and the effect of previous or current psychotropic medication and/or psychotherapy on neurofeedback training success and brain network connectivity. A follow‐up study to this PPI analysis should investigate the directed connectivity of the PCC during rt‐fMRI‐NFB using a generative modeling approach, such as dynamic causal modeling. Additionally, as most previous rt‐fMRI‐NFB studies have included only a single session of training, future studies should investigate PCC downregulation across multiple sessions which would help to elucidate the optimal ‘dose’ of neurofeedback for the treatment of PTSD. Furthermore, there are subgroups of clinical presentations of PTSD, such as the dissociative subtype, which should be considered separately by ideally powered studies to determine whether they are associated with unique responses to neurofeedback training. Last, further research is needed to investigate the involvement of the posterior DMN (i.e., PCC) in emotion regulation which may represent a fruitful research avenue in clinical neuroscience and psychiatry. Indeed, in the present study, there are several plausible emotion regulation strategies (e.g., effortless awareness, cognitive reappraisal), which may be associated with increased control over the PCC. Future research studies should seek to further clarify the efficacy of specific strategies.

## 5 CONCLUSION

In summary, we found that rt‐fMRI‐NFB targeting PCC downregulation led to within‐ and between‐group differences in functional connectivity between the PCC and DMN and SN hubs during regulate as compared to view neurofeedback training conditions. While both the PTSD and healthy control groups showed PCC connectivity to the precuneus/cuneus within the posterior DMN during regulation, only the PTSD group demonstrated PCC connectivity with the anterior DMN (vmPFC, dmPFC) and the SN (posterior insula). Also, as compared to the control group, the PTSD group showed PCC connectivity to the amygdala—another SN hub that is also highly significant in PTSD psychopathology—during regulation. Moreover, linear regression analyses found positive correlations between psychiatric symptoms and PCC connectivity to several key DMN (dmPFC) and SN hubs (anterior insula, amygdala, VS/NAcc). Taken together, PCC‐targeted neurofeedback may help in recalibrating aberrant connectivity within the subcortical limbic system and limbic‐related cortical brain regions and may play a role in improved emotion regulation among individuals with PTSD during neurofeedback training.

## CONFLICT OF INTEREST

All authors declare no financial interests or potential conflicts of interest with regard to the current study.

### PEER REVIEW

The peer review history for this article is available at https://publons.com/publon/10.1002/brb3.2883

## Supporting information

Table s1: History of Trauma Exposure Based on the Life Events Checklist for DSM‐5 (LEC‐5) Supplementary Methods s2

## Data Availability

The data that support the findings of this study are available from the corresponding author upon reasonable request.
