# Supplementary material for: Posterior cingulate cortex targeted real‐time fMRI neurofeedback recalibrates functional connectivity with the amygdala, posterior insula, and default‐mode network in PTSD
Source: Brain Behav. 2023 Feb 15;13(3):e2883. doi: 10.1002/brb3.2883 (PMC10013955; doi:10.1002/brb3.2883)
Supplement: Supplementary file 1 — Table s1: History of Trauma Exposure Based on the Life Events Checklist for DSM‐5 (LEC‐5) Supplementary Methods s2 [file BRB3-13-e2883-s001.docx]

**Table s1**

*History of Trauma Exposure Based on the Life Events Checklist for DSM-5 (LEC-5)*

| **Event** | | **PTSD Group** | **Healthy Control Group** |
| --- | --- | --- | --- |
| 1. | Natural disaster (e.g. flood, hurricane, tornado, earthquake) | *n=*7 | *n=*2 |
| 2. | Fire or explosion | *n=*6 | *n=*1 |
| 3. | Transportation accident (e.g. car accident, boat accident, train wreck, plane crash) | *n=*10 | *n=*8 |
| 4. | Serious accident at work, home, or during recreational activity | *n=*8 | *n=*1 |
| 5. | Exposure to toxic substance (e.g. dangerous chemicals, radiation) | *n=*8 | *n=*1 |
| 6. | Physical assault (e.g. physical abuse, being attacked, hit, slapped, kicked, beaten up) | *n=*13 | *n=*4 |
| 7. | Assault with a weapon (e.g. being shot, stabbed, threatened with a knife, gun, bomb) | *n=*8 | *n=*2 |
| 8. | Sexual assault (rape, attempted rape, made to perform any type of sexual act through force or threat of harm) | *n=*9 | *n=*1 |
| 9. | Other unwanted or uncomfortable sexual experience (e.g. inappropriate touching) | *n=*9 | *n=*3 |
| 10. | Combat or exposure to a war-zone (in the military or as a civilian) | *n=*5 | *n=*1 |
| 11. | Captivity (e.g. being kidnapped, abducted, held hostage, prisoner of war) | *n=*1 | *n=*0 |
| 12. | Life-threatening illness or injury | *n=*3 | *n=*1 |
| 13. | Severe human suffering | *n=*4 | *n=*0 |
| 14. | Witnessing sudden violent death (e.g. homicide, suicide) | *n=*5 | *n=*0 |
| 15. | Witnessing sudden accidental death | *n=*5 | *n=*0 |
| 16. | Serious injury, harm, or death you caused to someone else | *n=*6 | *n=*1 |
| 17. | Neglect by a caregiver (e.g. not getting the emotional or physical things you needed growing up, such as love, support, food, clean clothes) | *n=*11 | *n=*3 |
| 18. | Any other very stressful event or experience | *n=*1 | *n=*3 |

**Supplementary Methods s2**

*Neurofeedback PCC downregulation analysis*

To evaluate PCC downregulation (i.e., neurofeedback success), we used rfxplot software^1^ to extract the event-related BOLD signal (peristimulus time histogram) from the PCC target sphere during the *regulate* and *view* conditions. Specifically, we extracted the event-related BOLD signal from individual peaks within the target sphere. Within rfxplot software, event-related BOLD signals are estimated using a condition-specific Finite Impulse Response (FIR) model which yields the average height of BOLD signals within a defined search volume and time window^1^. Here, we parcellated the BOLD signal into time bins of duration, T = 2 seconds, beginning at condition onset. The parameter estimate for each time bin of the FIR model is the same as the mean BOLD signal for that particular bin. Hence, in sum, the FIR model creates an event-related BOLD time course for each subject for each condition. For the final display (Figure 2), group averages for event-related BOLD responses were plotted using rfxplot software^1^.

*State changes in emotional experience*

We assessed states changes in emotional response (i.e., reliving and distress, as measured by RSDI subscales) to traumatic/stressful stimuli over the course of neurofeedback training. Given that this data was not normally distributed, we first computed nonparametric Friedman’s repeated measures ANOVAs for each group and each RSDI subscale. We used the Bonferroni multiple comparison correction approach using a statistical threshold of *p* < .01 (*p* < .05/5) for nonparametric ANOVAs. Using non-parametric tests for related samples (Wilcoxon signed-ranks test), we conducted paired comparisons between time points. Then, we used Mann-Whitney U tests to compare the reliving and distress state scores across NFB runs and between groups.

1. *Gläscher J. Visualization of Group Inference Data in Functional Neuroimaging. Neuroinform. 2009;7(1):73-82. doi:10.1007/s12021-008-9042-x*
